# Hysteria and Brain Tumors

**Published:** 1888-06

**Authors:** 


**Hysteria and Brain Tumors.** By MARY PUTNAM JACOBI, M. D. Pp. 215; 8vo. G. P. Putnam Sons. 1888.

This book is composed of a number of essays which the distinguished authoress has prepared during the past few years, and they are well worthy of publication. The following are the subjects of the chapters:

I. Some Considerations on Hysteria.

II. Tumors of the Brain.

III. Note on the Special Liability to Loss of Nouns in Aphasia.

IV. Case of Nocturnal Rotary Spasm.

V. The Prophylaxis of Insanity.

VI. Antagonism between Medicine and between Medicines and Diseases.

VII. Hysterical Locomotor Ataxia.

The article on "Tumors of the Brain" is very timely, since much interest has recently been developed in the subject of cerebral surgery.

In the chapter on the "Prevention of Insanity," some new views regarding the etiology are advanced. She states that in minds predisposed to insanity there is often, if not always, a marked deficiency of elasticity. An impression sinks and remains, and the mind cannot disengage itself. Such minds should be educated up to the ability to change their occupation frequently.

The typographical work is, as is usual with the Putnams, of an excellent character.

